# Northeastern Dental Association

**Published:** 1899-02

**Authors:** 


### NORTHEASTERN DENTAL ASSOCIATION.

(Continued from page 59.)

*Evening Session, 7.30.*

*Dr. Geo. A. Maxfield* (Holyoke, Mass.).—I was in hopes certain members would be here who are not, because I wanted to say a few things which I desired them to hear. I have no report to make, simply a few remarks in regard to the dental laws. Dr. Taylor, in his remarks this morning, said that if you will only spend money all the dental laws can be knocked into pieces, and he is simply a mouth-piece for hundreds of others. I cannot understand why. Did you ever think that these laws are drawn up by lawyers? Now, if you heard a body of lawyers talk about filling teeth, you would think they were "off their base." Yet we talk about laws which have been before the Legislature. Governors have examined them and submitted them to their legal advisers. It seems to me that if we consider this matter a few moments, we can see that there is a good legal existence for these laws. Now, why is there so much opposition talk in the dental profession? One thing, a great many probably advocate these laws, thinking them an aid to the dental profession. The first law passed by the Massachusetts Legislature was vetoed by Governor North, after the promise had been given to sign the bill. His reason was that he might benefit the few,—benefit the dentists. Now, the object of the law is to benefit the public. If it benefits the public, wherein is it going to benefit the dentists? It benefits the dentists by putting a different quality of men in the dental profession.

The National Association of Dental Examiners held a meeting in Washington last week. Probably some of you were interested and remember the controversy which came up a year ago when the Association of Dental Examiners seemed to take such strong ground in demanding what the colleges should do if they were to be acknowledged. The language used was strong, and naturally raised considerable opposition in dental colleges. The intention was to bring the colleges up to a higher standard, and when you consider how a great many of the colleges are conducted, how they are run, simply for money, you can very soon see the desire upon the Examiners' part to obtain a higher standard. In their desire to get students, the colleges are not particular about whether the men are qualified, if they only pay the money. There is in Chicago a dental college which is controlled by one man, who owns all of the stock of the college. With one or two exceptions, this is a college as well equipped as any in the country. A great many students have come from that college thoroughly qualified. Now, because it is so now, is it right to say that it will always be so? The dental college should not be a means for making money. The laws for examining students are very rigid in some States.

Now, as to the meeting in Washington. We had representatives from seventeen examining boards,—Connecticut, Delaware, District of Columbia, Illinois, Kentucky, Maine, Maryland, Massachusetts, Minnesota, Missouri, Nebraska, New Jersey, Rhode Island, South Carolina, Tennessee, Virginia, and Wisconsin. There were a large number who came at an expense of from one hundred to one hundred and fifty dollars. They gained no personal benefit from it. They came for the purpose of attending the meeting. The benefit would be in raising the standard of the dental profession. As we look about and see the number recently entered into the profession, and take up the papers and read the advertisements, it is discouraging, making the dental profession simply a trade. But when men go to such an expense for the simple aim of elevating the profession, it shows that some men are working at a great sacrifice in order to bring a better class of men into the profession.

You know the impossibility of enacting laws which will be alike in all the States, and to have a certificate of examination in one State accepted in another will be a difficult thing to bring about. It is the plan of the Association to have all the States adopt a uniform examination and have a uniform standard in all of them. Then we will modify our laws so that a certificate of one State will be accepted in another State without an examination.

*Dr. L. C. Taylor* (Hartford, Conn.).—I am placed in rather an awkward position. The matter of interstate legislation is causing a growing uneasiness throughout the dental profession. An effort is being made by our commissioners to bring about a state of affairs where dentists will be allowed to register from State to State, but, so far as I have learned, they dodge just far enough so that the courts will throw it overboard the moment it is put into the field. If a man has been placed upon the list of competent practitioners by one State, and the Constitution says we must recognize the judicial acts of all the States, why is he not entitled to be a legal practitioner in all the States? Every State in the Union pledges that none of its laws shall conflict in any way with the Constitution of the United States. When the commissioners look at the law passed by the State, and see the clause which conflicts with the higher authority, they are bound to weigh the whole matter justly and honestly. You cannot discriminate after the stamp of legality has been placed upon any practitioner. I am decidedly in favor of the law, and want to retain it, but just as sure as we get a trial in the United States Court on fair constitutional grounds, that law is bound to be thrown overboard. A gentleman told me yesterday of a man who, on coming into a certain State, said that he held a diploma and intended to practise. If they wanted to call the matter before the courts, they could. He is to-day practising in that State, and no man dare touch him. The time is coming when something will be done, because it is right, and right will triumph if given sufficient time.

*A Member.*—Dr. Taylor says he wants the thing done. How shall it be done?

*Dr. Taylor.*—It is left with the board in this State to make their own regulations. It is a simple matter for them to make that law. As it is now, it depends upon the construction put upon it. The trouble lies in the way it is construed.

*Dr. C. C. Barker* (Meriden, Conn.).—We are all aware of the disease; the thing is to prescribe for it. There is no doubt concerning the disease. It seems a great injustice that a man cannot go through the length and breadth of the United States and practise his profession. If a dentist is a dentist in one place, he ought to be in the whole country. We cannot legislate this thing here in the State of Connecticut. If we want the thing done, get down to some practical method. When a number of men get together they are apt to be conservative. I would like to see a resolution passed indicating the sentiment of the Association. If some one can think of some favorable method, I think it would assist us.

*Dr. D. Murlless* (Holyoke, Mass.).—This is a matter that interests me,—the matter of raising the standard of dentistry. In regard to the rule regulating practice, I think Dr. Taylor made a very good remark,—that a man should practise whether he was a college graduate or not, provided he was legally qualified. Any man who practised before the law was enacted is entitled to practise now.

*Dr. Flanagan* (Springfield, Mass.).—This reminds me of a quotation: "The fellows who revile most against religion almost never set up a religion of their own." I see no reason why each and every man in his respective State should support the dental law. Let him stand by his position. When a thing is proved a failure, let him start out with the rest. I have known men who would like to see the law tried. They knew it could be broken. When the time came, they were anxious to get on the State Board and get on the other side. Those are not the men we want on the State Board. We want those who are willing to do something for dentistry. Every one is not willing to spend one hundred or one hundred and fifty dollars, but those who do are certainly on the right track.

Dr. L. B. Palmer read a paper on "The Relation of Science to Progress in Dental Practice." (For Dr. Palmer's paper, see page 78.)

The President announced as the Committee on Interstate Law, Dr. James McManus, Dr. W. H. Ryder, Dr. N. Morgan.

Dr. C. T. Stockwell opened discussion on the paper.

DISCUSSION.

*Dr. Stockwell.*—I am sure that we have, all of us, listened to Dr. Palmer's paper with great interest and appreciation. Because of it, notwithstanding what may have come before it, or whatever may follow it, this year's session of our society can be pronounced a success.

In attempting to fulfil the task assigned me on the programme, I find myself regretting exceedingly that I am not better acquainted with the science with which the essayist's name is so intimately associated. For twenty-five years, or longer, Dr. Palmer's name has been a household word in our profession. But who of us has given the matter, with which he has for so long a time so earnestly dealt, the attention it deserves?

Perhaps I may ask, Who of us is able, who of us is so well qualified, to deal with it as he has done?

There must be pioneers in every branch of science, I suppose; and certainly, in this respect and along this line of observation and investigation, Dr. Palmer is, in a conspicuous sense, a pioneer. In the closing page of his paper to-night he tells us that this is probably his last contribution to the profession on this subject. The question, then, may well arise, Is it not time for the profession to inquire a little more carefully than it has done heretofore with reference to the scientific basis of Dr. Palmer's presentations?

I must agree with Dr. Palmer that results obtained from laboratory experiments are not to be accounted sufficient to upset all clinical observations made at the chair. Furthermore, I also agree with him that there is a vital difference between experiments based solely on the physical plane and experiments and observations based upon the animal plane. The point made by Dr. Palmer in this respect seems to me a very important one, and will have to be taken into consideration by those who would get at all the facts.

Most of you here know, I presume, that I had something to do with the introduction of the germ theory of dental caries. I still hold, essentially, to that theory. That is, I hold, as I did ten years ago, that micro-organisms are an *essential factor* in dental caries. That without them there would be no caries. Still, I find myself in these later years coming to the conviction that the last word has not been said relative to the etiology of dental caries. The germ theory will, in my opinion, stand, as far as it goes. But it needs to be *supplemented* by other forces or energies not yet, perhaps, fully understood. Dr. Black has, for three or four years past, been working along certain lines now familiar to all. Dr. Palmer, for a score or more of years, has been prosecuting laborious and persistent scientific study along another line. In their conclusions they seem to differ somewhat materially. But I am led to ask myself if the difference is not more seeming than real. Dr. Black holds that a tooth's *liability* to decay does not lie in its *physical constitution*, but in its *environment*. In this environment Dr. Black's chief attention is fixed upon his plaques of microbes and the acids which they produce. I do not understand that Dr. Palmer denies the existence of these microbes and their consequent acids. Now, why may not the electric conditions which Dr. Palmer contends for constitute an essential and vital part of the environment which Dr. Black holds as solely responsible for dental caries? I do not see really, I frankly confess, why a so-called "soft" or "hard" tooth should not be acted upon with equal readiness by the "solvent of its lime-salts, an acid elaborated by micro-organisms." Its physical structure, alone and simply, should not, it seems to me, make any difference in regard to its susceptibility to the action of acids held against it by the presence of the mass on plaques which Drs. Black and Williams seem to have demonstrated. So, while it appears to me that Dr. Black's main contentions are likely to stand the test of further investigations,—I mean in so far as the physical structure of teeth is concerned,—I cannot accept his conclusions when he says, "With our *present knowledge*, the *only basis* for the selection and adaptation of filling-materials to classes of cases is the individual operator's judgment as to which *he* can so manipulate as to make the most perfect filling, considering the circumstances, his own skill, and durability of materials."

If this statement is to be accepted as final, then, as Dr. Palmer has stated, there is no science in filling teeth. There is a good deal more involved in the successful filling of teeth than the mere mechanical adaptation of materials used. The judgment and clinical experience of every thoughtful dentist of ten years' experience must, it seems to me, convince him of the truth of this statement.

Dr. Palmer's theory, as expounded here to-night, relates chiefly, if not entirely, to the recurrence of decay about gold fillings made in the teeth of young people. And here, it seems to me, he is thoroughly scientific. He is dealing with a factor—the electric conditions of the oral cavity—which some time in the future the profession will understand. For the last decade the world has been, in a practical way, entering a stage that is appropriately called an electric age. To my mind we are as yet in its infancy. Dr. Palmer is one of the pioneers—perhaps, I should say, *the* pioneer —in the study of the science of electricity as applied to dentistry. And I will confess to being no prophet if the day does not come when the profession does not recognize the fact that they owe to Dr. Palmer a great debt of gratitude.

I am not disposed to discuss the paper to any great extent. In fact, there seems to me little room for discussion. I can only urge every one of you to read and reread it very carefully when it shall appear in print. It will bear it. It is worthy of it. I say this very frankly and very gladly. I consider it a magnificent presentation of the relations of science to the progress of dentistry. It is entirely scientific in its matter and method, eminently professional, and profoundly ethical in its spirit and temper. And if, as intimated in his closing remarks, this is, indeed, to be his last utterance on this subject, it is in every way the fitting word, and one also that may well constitute an appropriate memorial of a work for humanity, which has absorbed his attention and been the devotion of his life for at least the last twenty years. And more than this, I am sure that I but voice the feeling of the members of the Northeastern Dental Association when I say that they esteem it a special honor and privilege to be the vehicle by which Dr. Palmer contributes this last word on this subject to the dental world.

Just a thought, in closing, on an allied subject. Dr. Palmer has spoken to us on the relations of science to the progress of dentistry. I have been impressed this evening as seldom, if ever, before with the thought of the relations of scientific men to the progress of dentistry. I refer now, specifically, to a class of men belonging to our own profession, men who are, in the truest sense, scientific, men whom any special branch of science would be proud to own and acknowledge. Their methods are scientific, their work is exact, profound, unselfish, and strictly devoted to the acquisition of the truth. The bald heads and gray beards of this audience, by letting their minds revert to the past twenty-five, thirty, or forty years, will recall the names of a few such men. I need not attempt their enumeration. Suffice it to say that they constitute a class of whom the essayist, this evening, is a conspicuous and illustrious example and type. Real scientific men are modest, unassuming, unpretentious. They do not make a great noise in the world,—quite unlike a larger class who vainly attempt to seize the livery of science in order to ride easily into prominence and temporary popularity. Of such men I am not now speaking. I refer only to those who are content to be martyrs, if need be, rather than to seek to be on the popular side of things and to go with the currents which make for commercial ends and personal aggrandizement. I am thinking now of men who are content to work unceasingly, to plod on in their quiet way, studying, observing, experimenting, and re-experimenting with a patience and a perseverance, year after year, that is wholly sublime, if not actually divine. And for what? Is it with and for a purpose that can be measured by the money standard, or anything that is implied by such a standard, such as houses and land, the ease and comforts and gratifications of a luxurious life? No; none of these things constitute the animating, moving purpose of these men's lives. But, rather, they burn the midnight oil, they toil, they spend lavishly from all their resources, in order that some law may be discovered, some fact may be demonstrated, which may lead to a truth or truths that shall be helpful in their own work and of advantage to their fellow-men.

I know of but one thing in the world that is better than science, and that is service. If any one chooses to ask me if religion is not the one thing better than science, my reply would be to ask such a one if service is not religion?

I have said, and said truly, that Dr. Palmer embodies, in his own being, this scientific spirit. He also, as his long life has shown, embodies the spirit of service. Who of us, having been in the practice of dentistry for the last quarter of a century, has not felt the tentacles of his searching but helpful spirit?

Some wise philosopher has said that "we have only that which we have given." Regardless of the fact of any other world than this, I believe that, in the deepest sense and perception of things, the philosopher is right. So believing, I congratulate Dr. Palmer and all like scientific men upon their great store of personal wealth,—wealth, not riches,—and acknowledge with the deepest gratitude my obligations to them.

I have been moved to say these things because we as a profession are too apt to be grossly unmindful of our obligations to such men as Dr. Palmer. We are too apt to forget that it is because of such men and their unselfish work that we possess any claim to be considered a profession at all. They have labored, and they labor unceasingly; and we, the great mass of the profession, receive, too often forgetful of the great debt we owe them, freely of the results of their labor.

Let us, then, on this auspicious occasion, at least make our guest feel we *can* be, and *are*, grateful for his earnest, scientific, and long-continued work in behalf of truth, the results of which he has so freely given to whomsoever hath an ear or an eye for knowledge.

Dr. S. S. Stowell, of Pittsfield, Mass., then read the following short practical paper.

(To be continued.)

